# Impact of Lipid Nutrition on Neural Stem/Progenitor Cells

**DOI:** 10.1155/2013/973508

**Published:** 2013-10-23

**Authors:** Nobuyuki Sakayori, Ryuichi Kimura, Noriko Osumi

**Affiliations:** ^1^Division of Developmental Neuroscience, United Centers for Advanced Research and Translational Medicine (ART), Tohoku University Graduate School of Medicine, 2-1 Seiryo-machi, Aoba-ku, Sendai 980-8575, Japan; ^2^Japan Society for the Promotion of Science, 8 Ichiban-cho, Chiyoda-ku, Tokyo 102-8472, Japan

## Abstract

The neural system originates from neural stem/progenitor cells (NSPCs). Embryonic NSPCs first proliferate to increase their numbers and then produce neurons and glial cells that compose the complex neural circuits in the brain. New neurons are continually produced even after birth from adult NSPCs in the inner wall of the lateral ventricle and in the hippocampal dentate gyrus. These adult-born neurons are involved in various brain functions, including olfaction-related functions, learning and memory, pattern separation, and mood control. NSPCs are regulated by various intrinsic and extrinsic factors. Diet is one of such important extrinsic factors. Of dietary nutrients, lipids are important because they constitute the cell membrane, are a source of energy, and function as signaling molecules. Metabolites of some lipids can be strong lipid mediators that also regulate various biological activities. Recent findings have revealed that lipids are important regulators of both embryonic and adult NSPCs. We and other groups have shown that lipid signals including fat, fatty acids, their metabolites and intracellular carriers, cholesterol, and vitamins affect proliferation and differentiation of embryonic and adult NSPCs. A better understanding of the NSPCs regulation by lipids may provide important insight into the neural development and brain function.

## 1. Introduction

Neural stem/progenitor cells (NSPCs) are a specific population of cells in nervous system that has self-renew capacity and multipotency. In early brain development, NSPCs divide symmetrically and increase their number to produce sufficient NSPC pool. Subsequently, an NSPC divides asymmetrically to produce one NSPC and one differentiated cell in an orderly fashion [1]. NSPCs at the early developmental stage generate a large amount of neurons, whereas those at the late developmental stage generate mainly glial cells [2]. New neurons migrate out and form synapses with other neurons, establishing neuronal networks, which are supported by glial cells including astrocytes and oligodendrocytes. The fact that all neurons and glial cells consisting the adult nervous system originate from NSPCs shows no doubt about the importance of these NSPCs in brain development.

Neurogenesis was traditionally considered to finish just after birth, although the possibility of neurogenesis in the adult rat brain is suggested already in 1960s [3, 4]. After a few decades of doubt against adult neurogenesis in mammals, Reynolds and Weiss found that cells dissociated from adult mouse brains proliferate to form spherical balls in culture [5]. These spherical balls are called “neurospheres” and are positive for nestin, a marker for NSPCs [6]. Neurospheres can differentiate into neurons, astrocytes, and oligodendrocytes following the withdrawal of growth factors from the culture medium. Clonal culture eventually confirmed self-renewal capacity and multipotency of these spheres [7]. When cells from a tissue form spheres *in vitro*, the original tissue is retrospectively considered to contain NSPCs. This selective culture of NSPCs for forming neurospheres is widely used in NSPC studies. Regarding *in vivo* studies, NSPCs are found as proliferating cells in the inner wall, subventricular zone (SVZ), of the lateral ventricle [8, 9]. It is further proven that cells in the SVZ that have features similar to astrocytes are NSPCs [10]. Regarding hippocampal neurogenesis, proliferating cells [11] and immature neurons [12] exist in the subgranular zone (SGZ) of the adult dentate gyrus. Late 90s is an epoch-making period that multiple papers are published from various laboratories showing the existence of actively proliferating NSPCs in the SGZ in rats [13] and mice [14], of adult-born neurons in the monkey brain [15] and in the postmortem human brain [16]. From these lines of evidence, it is now widely believed that NSPCs exist not only in the embryonic brain but also at least in two areas of the adult brain: the SVZ of the lateral ventricle and the SGZ of the dentate gyrus in the hippocampus. The NSPCs in the adult brain continuously produce new neurons that have important roles in rodent behaviors (see below), suggesting their significance in brain function.

Lipids are an important nutritional composition because they have high calorific value, compose biological structures, and produce biologically active substances. Lipid is an ambiguous term, and there is no definition widely accepted. It is frequently defined as naturally occurring compounds that are insoluble in water but soluble in nonpolar solvents. However, such a definition is somewhat misleading because many substances that are regarded as lipids are soluble in both water and nonpolar solvents. Lipids are often categorized into simple lipid, compound (or complex) lipid, and derived lipid [17], although another categorization is also well accepted [18]. Simple lipid is an ester of fatty acids and alcohol, for example, fat and wax. Fat is stored in adipocytes and is believed to be used as energy source for all organs except for nervous system. Compound lipid is a lipid with more groups, including phosphoric acid or carbohydrate, for example, phospholipid and glycolipid, respectively. These compound lipids are components of cell membrane. Simple lipids and compound lipids are metabolized or hydrolyzed into derived lipids, for example, fatty acids, steroids, and fat-soluble vitamins. These derived lipids have strong bioactivity and regulate various biological functions.

**Figure 1:**
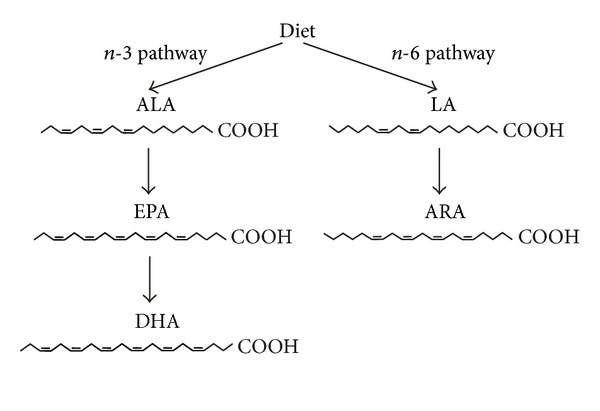
Synthesis of *n*-3 and *n*-6 PUFAs. DHA and ARA can be synthesized from ALA and LA, respectively.

Fatty acids are one of the derived lipids and behave as signaling molecules, precursors to families of lipid mediators, and components of both simple and compound lipids. In the brain, fatty acids are the major structural components; it is estimated that half of the neuronal membrane is composed of fatty acids [19–21]. Among fatty acids, long-chain polyunsaturated fatty acids (PUFAs), which have more than 16 carbon atoms and more than one *cis* double bond, have been implicated as critical nutritional factors for proper neural development and function [22–24]. Because the physiological properties of PUFAs largely depend on the position of the first double bond from the terminal methyl group of the carbon chain, PUFAs are categorized into *n*-3, *n*-6, and *n*-9 PUFAs by its position. They have the first double bond existing as the third, sixth, and ninth carbon-carbon bond from the methyl group, respectively. Most of lipids are synthesized *de novo* in mammals, while these *n*-3 PUFAs and *n*-6 PUFAs are not synthesized and must be obtained from diets [25]. Thus, *n*-3 PUFAs, including *α*-linolenic acid (ALA), eicosapentaenoic acid (EPA), and docosahexaenoic acid (DHA), together with *n*-6 PUFAs, including linoleic acid (LA) and arachidonic acid (ARA), are referred to as essential fatty acids. In a more rigorous definition, essential fatty acids are ALA and LA. This is because DHA and ARA can be synthesized from ALA through EPA and from LA, respectively (Figure 1). However, we should keep in mind that the synthesis of DHA from its original precursor, ALA, is very scarce in human [26]. The major end product of *n*-3 pathway is DHA, whereas that of the *n*-6 pathway in mammals is ARA. Actually, PUFAs in membrane phospholipids are mainly composed of DHA and ARA [25]. These fatty acids have indispensable roles in various biological functions.

Brain contains a large amount of lipids because neurons have very complicated dendrites and long axons that are ensheathed by the cell membrane of oligodendrocytes. There are many pieces of literature showing that lipids play pivotal roles in neural development and brain function because lipid is included in diet and affects as an extrinsic factor [27]. In this review, we focus on the effects of lipid nutrition in embryonic and adult NSPCs, mainly in rodents.

## 2. NSPCs and Their Function

### 2.1. Embryonic NSPCs in the Telencephalon

All neurons except for granule cells in the dentate gyrus and interneurons in the olfactory bulb (see below) are produced from embryonic NSPCs. All regions of the embryonic brain have NSPCs, and each character is slightly different. Here, we focus on embryonic NSPCs in the mouse telencephalon.

**Figure 2:**
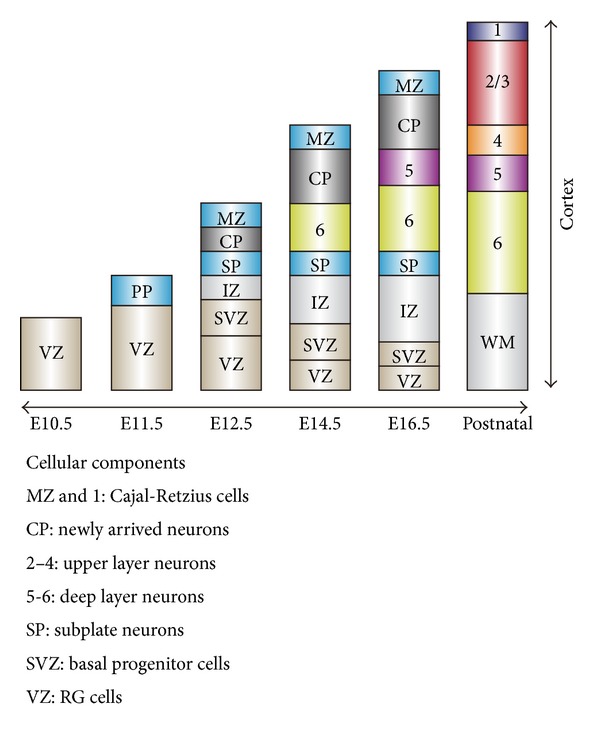
Neocortical development in the mouse. Preplate (PP) is composed of the earliest born neurons, which are differentiated from NSPCs in the VZ. PP is split into the subplate (SP) and marginal zone (MZ). NSPCs in the VZ also produce basal progenitor cells, resulting in the formation of the SVZ. The SP and MZ will form a part of layer 6 and the whole of layer 1 of the neocortex, respectively. Later, newborn neurons that form the cortical plate (CP) will form the multilayered neocortex in the postnatal brain, between layer 1 and layer 6. RG cells: radial glial cells, IZ: intermediate zone, and MW: white matter.

During early neural development, NSPCs emerge in the neural tissue at embryonic day (E) 8 in the mouse (or E10 in the rat). At this stage, NSPCs proliferate to expand the pool of NSPCs. Approximately at E10.5, NSPCs that reside in the inner wall of the neural tube start to produce cortical neurons. This region where NSPCs reside is termed the ventricular zone (VZ), and these NPSCs are called radial glial (RG) cells because their processes locate radially within the cortical primordium, and these cells exhibit astroglial properties [28]. RG cells also produce basal progenitor cells that further proliferate in the subventricular zone (SVZ) neighboring the VZ [29–31]. Neurons are produced by direct neurogenesis from RG cells and by indirect neurogenesis from basal progenitor cells [32]. Recently, another subtype of progenitor cells has been reported in the embryonic cortex. These progenitor cells are called outer radial glial (oRG) cells. oRG cells are generated directly from RG cells, form the outer subventricular zone (OSVZ), and produce neurons directly [33, 34]. oRG cells are implicated as an important source for cortical evolution because a recent study suggests that the development of oRG cells may be the cellular mechanism underlying expansion in primate corticogenesis [35]. The initial neurons produced from RG cells form the preplate, which is subsequently divided into the subplate and the marginal zone. The marginal zone will form layer 1 of the neocortex. From E11.5 to 17.5, RG cells, basal progenitor cells, and oRG cells produce projection neurons of the different neocortical layers in a strictly controlled temporal order, from layer 6 to layer 2/3 (Figure 2) [36, 37], although a recent report has shown that neuronal progenitor cells that will differentiate into upper layer neurons (layers 2–4) are already produced even in early neural development [38]. These neurons develop the cortical plate, which will give rise to the major layers (layers 2–6) of the gray matter of the neocortex, sandwiched by the subplate and the marginal zone. The production of cortical projection neurons is completed by the end of the embryonic period.

During late neural development, astrocytes are differentiated from NSPCs, and their production has its peak just after birth. Thus, there is a transition from neurogenic to gliogenic in the character of NSPCs. This transition is well studied *in vitro* because cultured NSPCs recapitulate the transition. NSPCs cultured for a short period differentiate into neurons, whereas those cultured for a long period produce more glial cells [2]. Moreover, the neurogenic-to-gliogenic fate switch of NSPCs can be observed in culture in clones of single NSPCs [2]. Several molecular mechanisms for the initiation/inhibition of astrocyte differentiation from NSPCs have been proposed [39–42].

Oligodendrocytes in the cortex are produced in three waves: the first and second waves occur in the embryonic ventral telencephalon, and the third wave occurs among postnatal cortical progenitors [43]. During embryonic development, oligodendrocyte precursor cells (OPCs) are thought to be generated from NSPCs located in the ventral telencephalon [43–45]. OPCs then migrate tangentially into the developing cortex [46, 47]. In addition to the production of embryonic OPCs, a postnatal wave of OPCs has been reported in the cortical SVZ [48, 49]. It is thought that a large portion of oligodendrocytes in the adult cortex originates from these OPCs [50]. OPCs differentiate into oligodendrocytes that form the myelin sheath surrounding neuronal axons. In some regions of the healthy adult brain, approximately 60% of OPCs continue to proliferate to generate oligodendrocytes [51]. Therefore, oligodendrogenesis is important throughout life.

### 2.2. Adult NSPCs in the SVZ of the Lateral Ventricle

**Figure 3:**
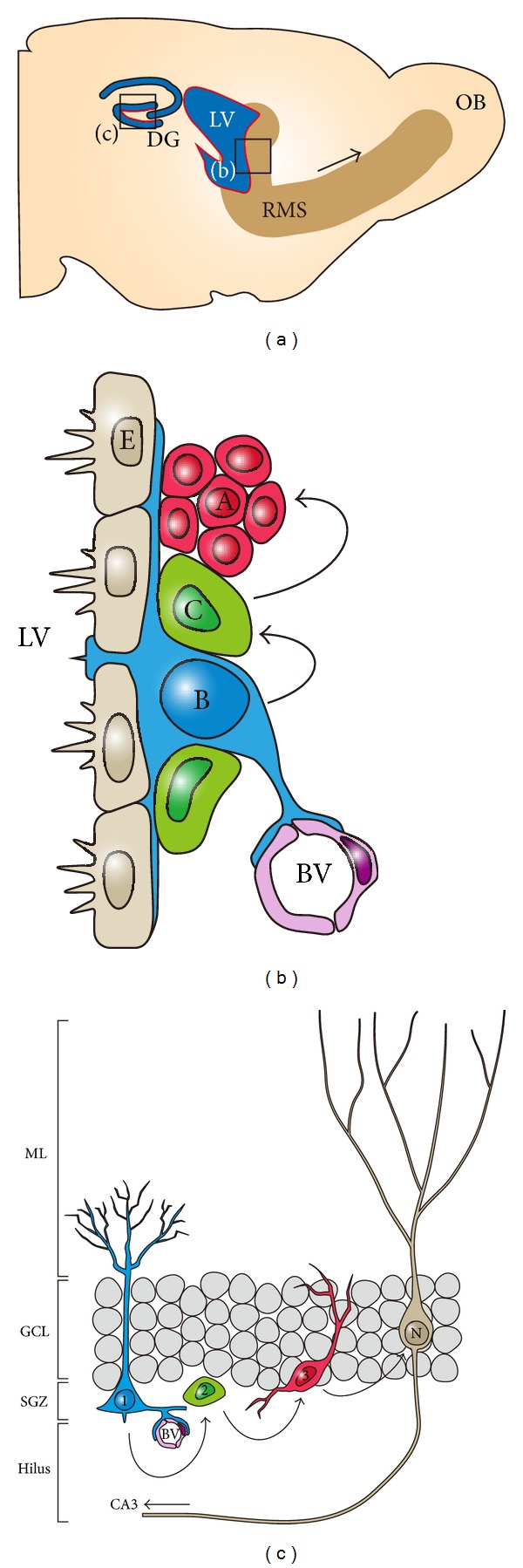
Adult neurogenesis in the rodent brain. (a) A sagittal view of the adult rodent brain. Red areas in the LV and DG indicate the SVZ and the SGZ, respectively, where active adult neurogenesis occurs. Arrow shows the direction of RMS through which immature neuroblasts born in the SVZ migrate to the OB. (b) A schematic image of adult neurogenic niche and sequential progression of adult neurogenesis in the SVZ. E: ependymal cell, A: type A cell, B: type B cell, C: type C cell, and BV: blood vessel. (c) A schematic image of adult neurogenic niche and sequential progression of adult neurogenesis in the SGZ. 1: type 1 cell, 2: type 2 cell, 3: type 3 cell, and N: mature neuron.

Several cell types are involved in adult neurogenesis in the SVZ (Figure 3). A lineage tracing study and fate-mapping study have revealed that GFAP-expressing cells that have morphological features similar to RG cells serve as quiescent neural stem cells [10, 52]. GFAP-expressing radial glia-like cells are referred to as type B cells [10]. These cells extend their small apical process that retains primary cilium to the ventricle. In addition, their basal processes reach blood vessels and form endfeet [53, 54], suggesting that type B cells are directly regulated by both cerebrospinal fluid and bloodstream. Type B cells give rise to actively proliferating progenitors, referred to as type C cells [10, 55]. Immature neuroblasts called type A cells are generated from type C cells and migrate a long way to the olfactory bulb (OB) through the rostral migratory stream (RMS) [8, 56]. Once type A cells reach the core of the olfactory bulb, they separate from the RMS and migrate radially toward the surface of the OB. Most of the type A cells become GABAergic granule neurons, but a minority of them become GABAergic/dopaminergic periglomerular neurons [57, 58].

Although the potential function of adult-born neurons in the OB is still under investigation, cumulative evidence has indicated their important roles in the OB functions. Half of the adult-born neurons in the OB are incorporated into the preexisting neural circuitry [59], and genetic ablation of newly generated cells in the SVZ resulted in a significant reduction in the number of mature granule neurons in the OB [60]. Many experiments have addressed the functional importance of adult-born neurons in olfactory-related behaviors. Although adult-born neurons in the OB are not required for the discrimination between similar chemical odors and response to innate aversive odor such as fox scent [60–62], they are required for olfactory-fear conditioning [62], olfactory perceptual learning [63], and long-term olfactory memory [64]. A recent study has demonstrated that adult-born neurons affect the response of mice to innate aversive odor when associated with reward [65]. Thus, adult-born neurons in the OB are important in olfaction-dependent behaviors via long-term structural integration.

### 2.3. Adult NSPCs in the SGZ of the Dentate Gyrus in the Hippocampus

The production and maturation of new granule neurons in the SGZ occur in a sequential manner as well as those in the SVZ (Figure 3). In the SGZ, GFAP-expressing radial glia-like cells are referred to as type 1 cells [66]. Type 1 cells extend their long apical process into the molecular layer (ML) of the dentate gyrus [52, 67], and their basal processes contact with blood vessels in the same fashion as type B cells in the SVZ [68]. Type 1 cells make a transition to fast proliferating intermediate progenitors called type 2 cells, which in turn generate neuroblasts (type 3 cells) [69]. Type 3 cells become immature neurons and migrate a short way into the inner granule cell layer (GCL), where they differentiate into granule neurons. Within two weeks, newborn granule neurons extend their dendrites toward ML and project their axon (mossy fiber) to CA3 pyramidal neurons through the hilus of dentate gyrus [70]. Compared to mature neurons, newborn granule neurons have hyperexcitability and enhanced synaptic plasticity during a certain period of time, contributing to shaping the existing circuit in response to external stimuli [71–73]. Newborn granule neurons gradually mature and work within preexisting neural circuit.

Adult neurogenesis in the SGZ has been implicated in several hippocampus-dependent behaviors. It has important roles in hippocampus-dependent learning and memory. Morris water maze task that is considered to activate the hippocampal neural circuit enhances the survival of newborn neuron in the dentate gyrus [74]. Irradiation or genetic manipulation of newborn neurons has also shown that adult neurogenesis in the SGZ is required for short- or long-term spatial memory [60, 75–77]. In addition to learning and memory, adult neurogenesis in the SGZ is also required for the formation of contextual fear memory and transition of such a kind of memory from the hippocampus to higher brain regions [78, 79]. Furthermore, other groups demonstrated that adult newborn neurons in dentate gyrus also contribute to the pattern separation by ablation with irradiation of adult neurogenesis in the SGZ [80] and by selectively inhibiting the synaptic transmission of old granule cells in dentate gyrus [81]. Pattern separation is a process to discriminate similar but distinct matters, and it is thought that the dentate gyrus and CA3 of the hippocampus play an important role in this process [82, 83]. Adult neurogenesis in the SGZ has also been implicated in mood control. Patients with major depressive disorder exhibit a reduced hippocampal volume, suggesting that decreased neurogenesis is one of the contributing factors [84]. In fact, antidepressant treatment in rodents and nonhuman primates increases neurogenesis in the dentate gyrus, and ablation of newborn neurons by irradiation attenuates the efficacy of antidepressant such as imipramine and fluoxetine on behavior [85–87]. Although there is criticism regarding the association between depression and neurogenesis (reviewed by Petrik et al. [88]), a recent study indicated that newborn neurons in the dentate gyrus are required for buffering the stress response through hypothalamo-pituitary-adrenal axis (HPA-axis) [89]. It seems that neurogenesis in the adolescent stage may contribute to the establishment of sensorimotor gating in the rat [90] and in the mouse (our unpublished results). Thus, various hippocampal functions are indeed at least in part related to newborn neurons in the SGZ.

Adult neurogenesis in the SGZ is well understood because it is regulated by lots of physiological stimulations. Physical exercise such as voluntary running enhances cell proliferation in the SGZ, and enriched environment promotes the survival of newborn neurons [14, 91]. On the other hand, various stress paradigms, for example, subordination, resident intruder, restraint, and isolation stresses, decrease cell proliferation in the SGZ [15, 92–94]. Aging also decreases in cell proliferation and neuronal differentiation in the SGZ [13]. A recent study has shown that age-associated decline of neurogenesis in the SGZ is attributable to depletion of neural stem cells followed by their differentiation into astrocyte [95]. Further study will reveal flexible characters of adult NSPCs in the SGZ and provide us with an insight into the regulation of stem cell activity in the adult tissue.

## 3. Modulation of NSPCs by Lipid Nutrition

NSPCs are regulated by various intrinsic and extrinsic factors. Intrinsic factors including genetic networks are difficult to manipulate. However, diet is one of the important extrinsic factors that can be easily manipulated. Here, we review nutritional effects of lipids on NSPCs.

### 3.1. Fat

Fat is a major dietary source for lipids and has significant involvements in NSPCs. Dietary fat is called triglyceride because it is a triester of glycerol and fatty acids. In triglyceride form, fat cannot be absorbed by the intestines. Pancreatic lipase hydrolyses the ester bond and releases fatty acids from glycerol. These derivatives can be absorbed and used by various organs. It is reported that obesity-inducing high-fat diet (HFD), when administered to mother mice, impaired the proliferation of early postnatal NSPCs but not of embryonic and young-adult NSPCs, in the hippocampus of their offspring [96]. Interestingly, another report has shown that HFD caused impairment of the proliferation of adult NSPCs in the SGZ without causing the apparent obesity [97]. These studies suggest the possibility that excess intake of fat is detrimental in NSPCs.

### 3.2. *n*-3 and *n*-6 PUFAs

Various *in vitro* studies have shown that *n*-3 and *n*-6 PUFAs are involved in the regulation of NSPCs. Previously, we have shown that DHA and ARA affect proliferation and differentiation of embryonic NSPCs [98]. We assayed embryonic NSPCs by neurosphere culture in DHA/ARA-free medium with/without DHA or ARA. For neurogenic NSPCs, DHA and ARA promoted the maintenance of NSPCs, but no detectable effects on differentiation were observed. For gliogenic NSPCs, DHA promoted the maintenance and neuronal differentiation of gliogenic NSPCs. Conversely, ARA did not promote the maintenance of NSPCs but promoted differentiation into astrocytes. We also confirmed that higher concentration of DHA had more toxic effects on the survival of NSPCs compared with that of ARA. This makes sense because DHA has more double bonds than ARA, and lipid peroxidation is a form of oxidative stress, which is toxic to cells and dampens cell survival [99]. These results show that DHA and ARA directly regulate embryonic NSPCs and that the effects of DHA and ARA on embryonic NSPCs depend on the stage of development. Other groups have also shown that DHA promotes the proliferation and neuronal differentiation of cultured NSPCs generated from embryonic stem (ES) cells [100] and that DHA induces the neuronal differentiation of cultured embryonic NSPCs [101–103]. Kan et al. found that both DHA and ARA are necessary for the neuronal differentiation from mesenchymal stem cells [104], suggesting that ARA may also be necessary for neuronal differentiation under some conditions.

The precursors of DHA and ARA, that is, ALA and LA, also affect NSPCs *in vitro*. We have previously shown that ALA and LA promote the maintenance of embryonic NSPCs [105]. On the other hand, it is also reported that conjugated linoleic acid (CLA), a positional and geometrical isomer of LA, promotes the neuronal differentiation of embryonic NSPCs, while LA has no such effect [106]. DHA and ARA may be synthesized in these experiments because embryonic NSPCs express enzymes that are necessary for the synthesis of DHA and ARA from ALA and LA [105]. It is possible that these enzymes regulate the metabolism of *n*-3 and *n*-6 PUFAs in the developing brain to regulate proliferation and differentiation of embryonic NSPCs.

*n*-3 and *n*-6 PUFAs actually affect NSPCs *in vivo*. We have previously shown that, by feeding DHA-rich diet to mother rats, there were no detectable effects on the proliferation of postnatal NSPCs in the SGZ of their offspring. However, it is reported that DHA is actually incorporated into the brain of offspring via the mother's breast milk [90], and another group has found that oral administration of DHA promoted adult neurogenesis in the hippocampus of rats fed with a fish oil-deficient diet over three generations [101]. It is also reported that by feeding *n*-3 PUFAs-rich diet to aged rat, immature neurons in the dentate gyrus was increased [107]. This is because age-related decrease of phospholipids [108] may partially be compensated by feeding *n*-3 PUFAs-rich diet. It is also known that feeding an *n*-3 PUFAs-deficient diet to pregnant rats causes inhibition or delay of neurogenesis in the embryonic brains of pups [109]. Regarding the effects of ARA, we have previously shown that supplementation of ARA to rat pups through mother's breast milk by feeding ARA-rich diet to mother rats promotes the proliferation of postnatal NSPCs in the SGZ [90]. These data suggest that DHA is necessary but not sufficient for regulating NSPCs in physiological condition but that ARA is sufficient to affect NSPCs even in the physiological condition.

### 3.3. Metabolites of *n*-6 PUFAs

Like *n*-6 PUFAs, their metabolites also influence NSPCs. *n*-6 PUFAs are metabolized into various substances [105, 110], including prostaglandins (PGs). PGs are strong lipid mediators and are known to have various functions in the regulation of NSPCs. E-type prostaglandin 2 (PGE_2_) is synthesized from ARA by cyclooxygenases (COXs) and microsomal PGE synthase-1 and functions by binding to PGE_2_ receptors, EP1 to EP4. EP3 is expressed in adult NSPCs [111, 112], and an EP3 agonist promotes the proliferation of adult NSPCs in the SGZ [113]. D-type prostaglandin 2 (PGD_2_) is also synthesized from ARA by COXs and two types of PGD synthase and is nonenzymatically metabolized into 15-deoxy-Δ^12,14^-prostaglandin J_2_ (15d-PGJ_2_), which also promoted the proliferation of cultured embryonic NSPCs and postnatal NSPCs in the hippocampus [114]. The fact that PGD_2_ is the most abundant PG in the brain [115] suggests the importance of 15d-PGJ_2_ function in NSPCs. Thus, mediators derived from ARA have significant roles in the regulation of NSPCs.

### 3.4. Fatty Acid Binding Proteins

Fatty acids taken from diets are delivered to various organs, but fatty acids need to be bound to proteins within the aqueous cytoplasm and blood plasma. This is because the solubility of fatty acids in aqueous solution is extremely low. Albumin can facilitate PUFA transport in blood plasma [116], and fatty acid binding proteins (Fabps) are intracellular carriers that accommodate PUFAs [117]. Among Fabps, Fabp3 (H-Fabp), Fabp5 (E-Fabp, K-Fabp, or S-Fabp) and Fabp7 (BLBP or B-Fabp) are the members expressed in the brain. Fabp3 is not expressed in the embryonic brain but appears in the adult brain [118]. Fabp5 is expressed in NSPCs in the embryonic brain and in the SGZ of the dentate gyrus in the hippocampus as well as in neurons in the cerebral cortex and in astrocytes [119–121]. Fabp7 is also expressed in NSPCs located in the VZ of the embryonic brain and in the SGZ of the dentate gyrus in the hippocampus and in astrocytes [121–124]. Among these Fabps, Fabp3 and Fabp5 bind to ARA [125, 126], while Fabp5 and more preferentially Fabp7 bind to DHA [127–129]. Due to these multiple Fabps, neural cells have access to various types of PUFAs at an adequate level.

The function of Fabps in the NSPCs has been studied by analyzing genetically altered mice. Fabp3 is not expressed in the embryonic brain and in the adult NSPCs [118], suggesting no function in NSPCs. Fabp5 and Fabp7 are strongly expressed in the embryonic brain, but no detectable abnormalities are reported in the gross anatomy of the brain of *Fabp5* and *Fabp7* KO mice [120, 130]. However, we have previously reported that the proliferation of postnatal NSPCs in the SGZ was decreased in both *Fabp5* and *Fabp7* KO mice [121, 124]. More severely reduced proliferation of NSPCs in the SGZ is observed in *Fabp5/7* double KO mice [121]. In addition, acute knockdown of Fabp7 promotes precocious neuronal differentiation, suggesting that Fabp7 is necessary for the maintenance of NSPCs [131]. These data show that Fabps, the intracellular carriers of PUFAs, have important roles in NSPCs.

### 3.5. Cholesterol

Cholesterol is one of the well-studied steroids in nutritional research. Cholesterol is an essential structural component of the cell membrane and forms lipid rafts interacting with various proteins to generate specific cholesterol-based membrane microdomains. These domains are important in membrane traffic and signal transduction [132]. Cholesterol also serves as a precursor for the steroid hormones and bile acids and is also a component of lipoproteins, that is, carriers for various lipids. About 25% of unesterified cholesterol is concentrated in the CNS [133]; the brain and spinal cord are the organs that contain the most abundant cholesterol among all organs, suggesting their importance in neural functions.

Roles of cholesterol in the regulation of NSPCs are poorly understood despite its significant functions on synaptogenesis [134]. The amount of cholesterol dramatically increases during cortical development [135], and an apical plasma membrane protein, prominin-1, of embryonic NSPCs in the VZ directly interacts with membrane cholesterol [136], suggesting that cholesterol has important roles in neural development. Conditional ablation of cholesterol biosynthesis in embryonic NSPCs leads to angiogenesis by increased vascular endothelial growth factor (VEGF) expression in embryonic NSPCs [137]. This may be a mechanism to compensate for the ablation of endogenous cholesterol, suggesting that cholesterol has essential roles in NSPCs. Regarding the roles of exogenous cholesterol, it is reported that the proliferation of adult NSPCs in the SGZ is decreased followed by feeding a high-cholesterol diet without increasing calorie intake [138]. These data suggest that appropriate biosynthesis/intake of cholesterol is necessary for the integrity of NSPCs.

### 3.6. Fat-Soluble Vitamins

Fat-soluble vitamins including vitamin A and E are important nutrients and also regulate the conditions of NSPCs. Vitamin A is well known to be necessary for visual functions and regulation of some genes including *α*B-crystallin and fibroblast growth factor 8 [139]. Regarding their effects on NSPCs, it is reported that the injection of an excess dose of retinoic acid (RA), an active form of vitamin A, to mice significantly reduced the proliferation of adult NSPCs in the SGZ and SVZ, suppressed adult hippocampal neurogenesis, and disrupted the ability to perform a spatial radial maze task [140]. Depletion of RA in adult mice, on the other hand, leads to significantly decreased neuronal differentiation and reduced neuronal survival within the granular cell layer of the dentate gyrus [141]. RA can restore adult hippocampal neurogenesis in retinoid-deficient rats [142]. These data suggest that a suitable dose of RA is essential for NSPCs.

Vitamin E is a group of compounds with well-known antioxidant functions. Supplementation of *α*-tocopherol, the most important compound of vitamin E, inhibits the proliferation of adult NSPCs in SGZ, conversely promoting neurogenesis and enhancing the neuronal survival in the dentate gyrus [143]. On the contrary, vitamin E deficiency in rats causes increased proliferation of adult NSPCs in SGZ and reduced neuronal survival [144]. These data clearly show that vitamin E promotes neuronal differentiation of NSPCs and survival of neurons.

### 3.7. Confounding Factors in Nutritional Research

Not only nutritional contents but also calorie intake, meal frequency, and meal hardness do affect proliferation and differentiation of NSPCs. Restriction of calorie intake increases the numbers of newly generated cells in the dentate gyrus of the hippocampus as a result of increased cell survival [145]. Extending the time between meals without reducing calorie intake also increases adult hippocampal neurogenesis [146]. In addition, cell proliferation in SGZ is decreased followed by feeding powder diet compared to by feeding solid diet [147]. These parameters can be confounding factors that affect basal characters of NSPCs. There are more possible confounding factors that may affect NSPCs, including taste and smell of food, because these factors play important roles in regulating food intake. Researchers on nutritional studies should keep in mind these potential secondary effects.

## 4. Conclusions

Embryonic NSPCs are essential for neural development and adult NSPCs are important for various neural functions, including cognition and mood. It is now becoming clearer that lipid nutrition has a significant impact on neural development and brain functions. Modulating proliferation and differentiation of NSPCs by diet could be an easily controllable intervention that may prevent neurodevelopmental disorders, cognitive decline during aging, and various kinds of psychiatric disorders. Indeed, *n*-3 PUFAs have ameliorative/preventive effects on patients with schizophrenia [148–151], mood disorders [152–154], and posttraumatic stress disorder [155–157]. A recent report has shown that ARA may potentially have a therapeutic effect on autistic patients [158]. Although effects of lipid nutrition are well focused, mechanisms by which lipid nutrition modulates NSPCs are poorly understood. Fatty acids serve as ligands for several G-protein-coupled receptors. It is recently reported that one of such receptors, that is, GPR40, is necessary for DHA-inducing neuronal differentiation of embryonic NSPCs [103]. GPR40-dependent phospholipase activation may thus be a possible signaling pathway of DHA. Further studies are necessary for comprehensive understanding of the effects of lipid nutrition.
